# Annual Meeting of the Western Texas Medical Association

**Published:** 1898-11

**Authors:** 


### Annual Meeting of the Western Texas Medical Association.

The twenty-second annual meeting of the Western Texas Medical Association was held in Dr. B. F. Kingsley's offices in San Antonio, October 25, ult. There were present Drs. Louise Jarvis, A. S. Dupuy, Wm. F. James, J. P. Oldham, B. F. Kingsley, F. Paschal, E. Clavin, S. Burg, F. M. Hicks, R. E. Moss, J. S. Lankford, H. D. Barnitz, Wm. E. Luter, B. E. Hadra, A. C. McDaniel, C. D. R. King, R. L. Withers, L. L. Shropshire, J. H. Bell, H. Sanvignet, Jones, J. S. Turner, of the Southwestern Asylum, A. Garwood, of New Braunfels, H. Leonard, of New Braunfels, E. L. Sharp, of Pleasanton, C. W. Orr, of Kenedy, J. R. Evans, of Devine.

The following program was carried out:

Medical and Surgical Practice During the Santiago Campaign—By Dr. Wm. F. James, San Antonio.

Tubercular Arthritis and Report of Case—By Dr. Louise Jarvis, San Antonio.

Comparative Photo-Micrography as Applied to Medical and Scientific Research, Illustrated—By Dr. R. Menger, San Antonio.

Report of Cases—(1)—An extensive osteomy elitis of femur with operation. (2) A large abdominal hæmatoma and its evacuation, by Dr. Alfred C. McDaniel, San Antonio.

Internal Hemorrhage—By Dr. F. Paschal, San Antonio.

Retro Uterine Hæmatocele—By Dr. Sigmund Burg, San Antonio.

The election of officers resulted as follows: President, Dr. John S. Lankford, of San Antonio; first vice-president, Dr. S. Burg, of San Antonio; second vice-president, Dr. Louise Jarvis, of San Antonio; secretary and treasurer, Dr. Wm. E. Luter, of San Antonio; board of directors, Drs. H. D. Barnitz, J. H. Bell, F. Paschal, T. J. Largen, L. L. Shropshire.

All the papers read were interesting and instructive and the discussions were enthusiastic.

The paper of Dr. Wm. F. James elicited a great deal of interest and he exhibited specimens of the different kinds of bullets used and explained the character of wounds produced and methods of dressing and caring for the patients on the field of battle around Santiago.

After election of officers the retiring president, Dr. H. D. Barnitz, made a short address and adjourned the association to the banquet hall.—San Antonio *Express,* October 26.

